# Novel Insights into Somatic Comorbidities in Children and Adolescents Across Psychiatric Diagnoses: An Explorative Study

**DOI:** 10.1007/s10578-023-01587-w

**Published:** 2023-09-01

**Authors:** Jet B. Muskens, Wietske A. Ester, Helen Klip, Janneke Zinkstok, Martine van Dongen-Boomsma, Wouter G. Staal

**Affiliations:** 1 Karakter Child and Adolescent Psychiatry University Centre Nijmegen, Nijmegen, The Netherlands; 2 Department of Psychiatry, Radboud University Medical Centre, Nijmegen, The Netherlands; 3 Department of Cognitive Neuroscience, Donders Institute for Brain, Cognition and Behaviour, Radboudumc, Nijmegen, The Netherlands; 4 Sarr Autism Rotterdam, Youz Child and Adolescence Psychiatry, Dynamostraat 18, Rotterdam, 3083 AK The Netherlands; 5 Parnassia Psychiatric Institute, Kiwistraat 30, The Hague, 2552 DH The Netherlands; 6 Department of Child and Adolescent Psychiatry, Curium-LUMC, Leiden University Medical Center, Endegeesterstraatweg 27, Oegstgeest, 2342 AK The Netherlands; 7 Department of Psychiatry, University Medical Center Utrecht, Utrecht, The Netherlands; 8 Leiden Institute for Brain and Cognition, Leiden, The Netherlands

**Keywords:** Child and adolescent psychiatry, Somatic comorbidity, Physical health, Insomnia, Obesity, Vitamin D deficiency

## Abstract

Many children with psychiatric disorders display somatic symptoms, although these are frequently overlooked. As somatic morbidity early in life negatively influences long-term outcomes, it is relevant to assess comorbidity. However, studies of simultaneous psychiatric and somatic assessment in children are lacking. The aim of this study was to assess the prevalence of somatic comorbidities in a clinical sample of children and adolescents with psychiatric disorders in a naturalistic design. Data were assessed from 276 children with various psychiatric disorders (neurodevelopmental disorders, affective disorders, eating disorders and psychosis) aged 6–18 years. These data were collected as part of routine clinical assessment, including physical examination and retrospectively analyzed. For a subsample (n = 97), blood testing on vitamin D3, lipid spectrum, glucose and prolactin was available. Results of this cross-sectional study revealed that food intake problems (43%) and insomnia (66%) were common. On physical examination, 20% of the children were overweight, 12% displayed obesity and 38% had minor physical anomalies. Blood testing (n = 97) highlighted vitamin D3 deficiency (< 50 nmol/L) in 73% of the children. None of the predefined variables (gender, age, medication and socioeconomic factors) contributed significantly to the prevalence of somatic comorbidities. The main somatic comorbidities in this broad child- and adolescent psychiatric population consisted of (1) problems associated with food intake, including obesity and vitamin D3 deficiency and (2) sleeping problems, mainly insomnia. Child and adolescent psychiatrists need to be aware of potential somatic comorbidities and may promote a healthy lifestyle.

## Introduction

“The mind is the body and the body is the mind”. This statement that opposes the Cartesian dualism [1] is supported by the high co-occurrence and relationship between somatic and psychiatric conditions [2, 3]. In the current available literature there is not one unambiguously definition for somatic condition. Different terms are used for somatic conditions e.g. somatic symptoms, somatic health problems, somatic health issues, somatic concerns, somatic comorbidities, somatic risk factors, medical or physical conditions and health problems [2–4]. In this paper we define somatic conditions as risk factors for somatic illnesses later in life, including adverse effects of medication, addiction problems and lifestyle related factors (e.g. unhealthy dietary habits, unhealthy weight, disordered sleep).

Various studies, mainly based on the adult population, show that the pathways between psychiatric and somatic disorders are complex, bidirectional and share common risk factors [2]. Associations between somatic and specific psychiatric conditions are partially found in research investigating children. For example, children with a diagnosis of autism spectrum disorder (ASD) and/ or attention deficit hyperactivity disorder (ADHD) frequently display obesity and neurologic disorders while children with diabetes type I or asthma show mood and anxiety disorders [4–9] As such, increased awareness of the co-occurrence of somatic and psychiatric health issues is warranted [2–9]. However, little is known about the prevalence of somatic disorders in the daily child psychiatric practice.

Although many children with psychiatric disorders appear to display comorbid somatic symptoms, these are frequently overlooked [10–12]. In general, patients with psychiatric disorders are more likely to develop somatic comorbidities [2, 3]. Vice versa, somatic disorders may cause, enhance or camouflage psychiatric symptoms [2]. Of major concern is that most psychiatric disorders arise during childhood [13] and that many somatic comorbidities in children with psychiatric disorders continue into adulthood [12]. Therefore, it is crucial, from a preventive perspective, to investigate somatic risk factors and comorbidities in children with psychiatric disorders to develop guidelines that integrate physical and mental health to improve outcomes.

Most data on somatic comorbidities in child and adolescent psychiatry are confined to neurodevelopmental disorders (NDD), especially ASD and ADHD. A systematic review showed a broad range of somatic comorbidities in NDD, which can manifest across different somatic areas, such as immunology (asthma, atopy, food allergy), neurology (seizures and epilepsy) and gastroenterology (abdominal pain, nausea, malnutrition, constipation) [14]. The increased risk for neurodevelopmental disorders has also been found in children with somatic disorders. For instance, hospitalized children for somatic conditions in a pediatric care setting showed a higher prevalence of neurodevelopmental disorders compared to the general population [15]. This emphasizes the need to screen for neurodevelopmental disorders in hospitalized children with somatic conditions. In a primary care setting, children with ASD showed more feedings problems, enuresis, luxations and sleeping disorders and were more referred to physiotherapists, speech therapists and to ear/nose/throat doctors compared to children without ASD [16]. Unfortunately neurodevelopmental disorders are often not recognized in primary or hospital care. Nevertheless, as NDD may also indicate underlying genetic syndromes with an increased risk of somatic and psychiatric vulnerability, multidisciplinary healthcare services are recommended in these children [14].

In addition, the awareness that lifestyle factors may also relate to psychiatric disorders has grown rapidly [17]. Reviews indicate that an unhealthy lifestyle in patients with psychiatric diagnoses contributes to adverse somatic health outcomes (e.g. overweight and obesity, dyslipidemia, hypertension, hyperglycemia (metabolic syndrome), steatosis, constipation, cardiovascular diseases, type 2 diabetes mellitus) [2, 3, 18]. The reason for the increased risk of adverse somatic health outcomes in psychiatric patients is multifactorial and includes iatrogenic effects of medication, lifestyle factors as well as genetic and pathophysiologic effects [2, 3, 17, 18]. This also appears relevant for children with psychiatric disorders, although large-scale longitudinal studies of lifestyle factors in children with psychiatric diagnoses are sparse [18].

A recent register based study of children and adolescents showed associations between a wide range of psychiatric and somatic diagnoses across all types of conditions and across all ages [12]. However, ASD was not included in that study while several demographic variables, such as socioeconomic factors were not accounted for. Thus far, data of somatic comorbidities in children and adolescents with broader psychiatric diagnoses are relatively sparse and allow no firm conclusions due to limited numbers of participants and/ or a lack of robust methodological design [4, 19, 20]. As such, a clear picture about the impact of somatic comorbidities in child psychiatric services is missing.

Here, we performed an observational, cross-sectional cohort study to assess the prevalence of somatic comorbidities in a sample of children and adolescents with various psychiatric disorders using a standardized somatic assessment incorporated to the mental state examination. The somatic assessment entails a standardized questionnaire followed by an interview with the child and caregivers and physical and laboratory examination of the child. The aim of the somatic screening assessment was to explore and identify new or not yet recognized somatic conditions (such as sleep disorders, underweight/ overweight/ obesity, hypertension) in a naturalistic sample of children and adolescents with various psychiatric diagnoses. We hypothesize that somatic comorbidities are widespread in children and adolescents with psychiatric disorders.

## Materials and Methods

As part of an ongoing explorative assessment on somatic comorbidity and lifestyle in children and adolescents with psychiatric disorders, we retrospectively reviewed documents and medical records from patient files between March 2020 and December 2020, including physical and laboratory measurements at diagnostic assessment carried out between October 2017 and March 2020 at the child hospital of Karakter University Centre – Radboud University Hospital. The authors assert that all procedures contributing to this work comply with the ethical standards of the relevant national and institutional committees on human experimentation and with the Helsinki Declaration of 1975, as revised in 2008.

### Study Sample

The study sample included children and adolescents aged 6–18 with psychiatric disorders based on DSM-IV-TR and/ or DSM 5 (since 1 January 2018 used in the Netherlands) [21, 22]. Psychiatric disorders were classified into four categories based on primary diagnosis: (1) neurodevelopmental disorders (ASD, ADHD, Tourette syndrome), (2) affective disorders (unipolar depression, bipolar disorder, dysthymia/ persistent depressive disorder, disruptive mood dysregulation disorder, generalized anxiety disorder, obsessive-compulsive disorder, panic disorder, posttraumatic stress disorder, separation anxiety disorder, social phobia), (3) eating disorders (anorexia nervosa, bulimia nervosa) and (4) psychosis. Insomnia criteria are also based on DSM-IV-TR and DSM 5 criteria [21, 22]. Karakter UC provides psychiatric care for children and adolescents with an average or high intelligence level. Participants were required to have a minimum average estimated intelligence (IQ > 70). Estimated intelligence was based on either clinical functioning (e.g., good functioning at school) or by assessing an intelligence test (Wechsler Intelligence Scale for Children, if available [23]). Children who were supplemented with vitamin D3 at the moment of assessment were excluded from the study (n = 3). These three children were excluded in the laboratory assessment because of supplementation with vitamin D3, but not excluded in the physical assessment. Two children were not diagnosed with a psychiatric disorder and were therefore excluded. Children with pre-existing somatic conditions or comorbidities, for instance asthma, atopy, epilepsy, were also assessed during the somatic screening and not excluded in the study. Pre-existing somatic conditions or comorbidities were not included in the data.

### Measures

#### Psychiatric Assessment

Prior to the first appointment, questionnaires were analyzed on child’s ethnicity, parent’s educational level and family history. Ethnicity of the child was based on the parental country of birth. If both parents were non-Dutch, mother’s ethnicity determined the ethnicity of the child. Parents’ educational level was determined by the highest completed education (primary school, secondary school and higher education).

During the first appointment, a psychologist and child and adolescent psychiatrist interviewed the child and caregivers. Next, a mental state and physical examination was carried out by the child and adolescent psychiatrist or a nurse practitioner, supervised by a child and adolescent psychiatrist. The standardized questionnaire and interview involved the somatic and psychiatric history of the child, current somatic symptoms, eating and sleeping habits, medication use and family history for psychiatric disorders (yes or no; first and second-degree family) and somatic disorders (familiar insults or epilepsy, familiar cardiovascular diseases, hypercholesterolemia and Diabetes Mellitus; known genetic syndromes or intellectual disabilities in family). Besides somatic symptoms we also asked for drugs/ alcohol use, history of trauma and serious life events (SLE). SLE in this study is defined as divorce of caregivers, death of caregiver, serious illness to family members, physical and/or sexual abuse, bullying at school and living in foster care. The structured interview was followed by a physical and laboratory assessment.

The psychiatric diagnosis was made by a structured interview by the psychologist, child and adolescent psychiatrist and nurse practitioner and based on DSM-IV-TR and DSM 5 criteria [21, 22]. Consensus diagnoses were established after the assessment by a multidisciplinary team consisting of two independent child and adolescent psychiatrists, psychologists and two nurse practitioners. The psychologist, child and adolescent psychiatrist and nurse practitioner who met the children during the first appointment were part of this multidisciplinary team.

#### Physical and Laboratory Assessment

In this study somatic conditions were assessed during physical examination of the child/ adolescent by a child and adolescent psychiatrist or nurse practitioner (supervised by a child and adolescent psychiatrist). Physical examination included measurement of weight, height, blood pressure, heart rate, inspection of minor physical anomalies (MPA) and signs of non-self-injury behavior. MPAs are subtle, abnormal morphological features, such as deviations in morphology of the head, eyes, ears, mouth, hands, and feet [24]. MPA were mentioned in the results when at least three dysmorphic features in a child were present based on recommendation by a clinical geneticist and evaluated in a previous study [25]. Non-self-injury behavior (NSSI) consists of behaviors such as self-cutting, scratching, hitting or banging, carving, and scraping often seen as inadequate coping behavior to regulate overwhelming distress [26].

The prevalence of children being underweight, overweight or obese was defined by the cut-off values of BMI references per age group according to the International Obesity Task Force [27]. Blood pressure was measured with an oscillometric device, widely used to screen initial blood pressure in a clinical setting. Persistent childhood hypertension (HTN) was diagnosed when repeated blood pressure was measured above the 95th percentile for the age, gender and height of the patient or above 130/80 mmHg on three separate visits.

Blood testing included vitamin D3, glucose, prolactin levels and lipid profiles. Indications for blood testing were based on available clinical guidelines [28] from the Dutch Knowledge Centre for Child and Adolescent psychiatry and the consensus statement of the American Diabetes Association, the American Psychiatric Association, the North American Association for the Study of Obesity, and the American Association of Clinical Endocrinologists in recommending metabolic screening and monitoring, for all patients receiving second generation antipsychotics, regardless of age [29]. Blood specimens were processed at a local laboratory from which references were used to interpret the results of the laboratory findings. For vitamin D3 references from the American Academy of Pediatrics Committee on Nutrition and The Institute of Medicine corresponded to a serum 25-hydroxyvitamin D level of at least 50 nmol/liter [30].

#### Data Analysis

Patient baseline characteristics were reported using frequency distributions for the categorical variables and mean ± standard deviation for the continuous variables when normally distributed. Otherwise, the median and interquartile range was reported. The prevalence of somatic comorbidities was presented as a frequency distribution for the different diagnostic groups and the total group. A fisher exact test was used if the expected cell frequencies were smaller than five. A p-value < 0.05 (two-sided) was considered statistically significant in descriptive statistics. Sub-analyses were done with different variables (gender, age, primary diagnosis, medication, and educational level of caregivers) to determine their influence on the results of the somatic examination and laboratory findings. All analyses were conducted using SPSS version 24 (IBM Corporation, Armonk, NY).

## Results

**Fig. 1:**
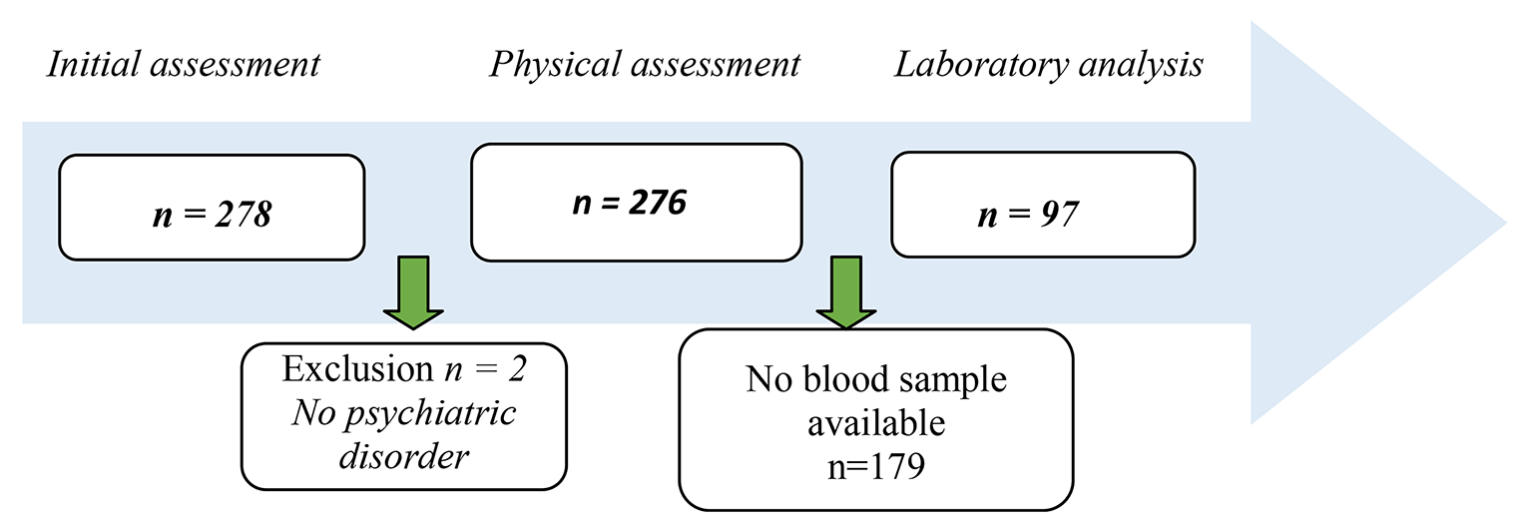
Flowchart of study inclusion of the study population

As shown in Fig. 1, 278 children were referred to the psychiatric hospital in the previously stated time frame. Two were not diagnosed with a psychiatric disorder and were therefore not included. Laboratory measurements were available for 97 children.

Table 1 presents the baseline demographic and clinical characteristics of the participants. Of the 276 patients, 145 were male (52.5%) with a mean age of 12.9 years (range 6–18, SD 3.5). A total of 93.1% of the children were of Dutch ancestry (North European), 3.6% of the children were adopted and 40.9% of parents had separated or divorced. In terms of parental education, 116 parents (42.0%) reportedly completed higher education, 104 parents (37.7%) completed secondary education only; and 39 parents (14.1%) completed primary education. Positive family history of psychiatric disorders was present in 65.6% of the children. Exposure to stressful life events of the child was conveyed by parents in 56.1% of the children. The two most common primary diagnoses were NDD (63%) and affective disorders (29%). In the NDD group, 24 children did have ADHD and 149 ASD as primary diagnosis; for affective disorders, 43 children did have a depressive disorder and 37 an anxiety disorder as primary diagnosis. A total of 149 children were treated with one type of psychotropic drug or a combination of drugs (54.0%) at the time of somatic assessment. None of the predefined variables (gender, age, medication and socioeconomic factors) contributed significantly to the prevalence of somatic comorbidities

**Table 1:** Description of the study population (n = 276)

		n	%
Age	6–11 year	88	31.9%
	12–14 year	72	26.1%
	15–18 year	116	42.0%
Gender	Male	145	52.5%
	Female	131	47.5%
Stressful life events	Yes	155	56,2%
	No	121	43,8%
Primary psychiatric diagnosis	Neurodevelopmental disorder	173	62.7%
	Affective disorder	80	29.0%
	Eating disorder	12	4.3%
	Psychosis	11	4.0%
Psychotropic medication	Yes	149	54,0%
	No	127	46,0%
	If yes,		
	Antipsychotics	96	64.4%
	Stimulants	35	23.5%
	Antidepressants	17	11.4%
	Mood stabilizers	1	0.7%
Educational level primary caregivers	Primary education	39	14.1%
	Secondary and lower vocational education	104	37.7%
	University degree, higher professional education	116	42.0%
	Information is missing	17	6.2%
Family history of psychiatric diagnosis	Negative	90	32.6%
	Positive	181	65.6%
	Information is missing	5	1.8%

Table 2 presents the somatic comorbidities for the total group and the four primary psychiatric diagnoses groups. Questionnaires and somatic interviews of the child and caregivers displayed insomnia (n = 182; 66%) and problematic food behavior, including selective eating, food neophobia, limited intake or hyperphagia (n = 119; 43%) as major complaints in the total group. Of note, insomnia frequency rates were high for all psychiatric diagnoses (55–91%).

The physical assessment indicated that 19.9% of the children were overweight and 12.0% obese, according to BMI. Overweight and obese ranges were similar across psychiatric diagnoses (overweight 20–27%; obese 12–18%), except for children with eating disorders. For children using antipsychotic medication, 25.0% of the children were overweight and 13.5% obese. Minor physical anomalies were present in 52.6% of the children with NDD and in 54.5% of the children with psychosis, compared to 8.8% in children with affective disorders and absent in children with eating disorders. Signs of non-self-injury behavior were noticed in 44 children (16.1%).

Measurement of height (2 ≤ sd ≥ 2) was not significant between the different diagnosis groups. Prevalence rates of hypertension were low across all diagnostic groups and not significant. In total, the prevalence of childhood hypertension (repeated blood pressure measured on three separate visits) was 2.5% (7/276 children). The prevalence of hypotension (systolic < 90mmHg or diastolic < 60mmHg) was 4% (12/276).

Alcohol and/ or drug abuse was mentioned by 20 adolescents (7%; not significant).

**Table 2:** Primary diagnosis in relation to somatic comorbidity in the study population

		Total		NDD		Affective Disorder		Eating Disorder		Psychosis		p-value^*^
		(n = 276)		(n = 173)		(n = 80)		(n = 12)		(n = 11)		
		n	%	n	%	n	%	n	%	n	%	
Insomnia	Absent	94	34.1%	78	45.1%	15	18.8%	0	0%	1	9.1%	< 0.01
	Present	182	65.9%	95	54.9%	65	81.3%	12	100%	10	90.9%	
Eating behavior problems	Absent	157	56.9%	102	59.0%	48	60.0%	1	8.3%	6	54.5%	< 0.01
	Present	119	43.1%	71	41.0%	32	40.0%	11	91.7%	5	45.5%	
BMI category	Underweight^a^	35	12.7%	13	7.5%	8	10.0%	11	91.7%	3	27.3%	< 0.01
	Normal	153	55.4%	104	60.1%	45	56.3%	1	8.3%	3	27.3%	
	Overweight^b^	55	19.9%	35	20.2%	17	21.3%	0	0.0%	3	27.3%	
	Obese^b^	33	12.0%	21	12.1%	10	12.5%	0	0.0%	2	18.2%	
MPA	Absent	169	61.2%	79	45.7%	73	91.3%	12	100%	5	45.5%	< 0.01
	Present	104	37.7%	91	52.6%	7	8.8%	0	0%	6	54.5%	
	Missing	3	1.1%	3	1.7%	0	0.0%	0	0%	0	0.0%	
Signs of non-self-injury behavior	Absent	230	83.9%	153	89.5%	65	81.3%	6	50.0%	6	54.5%	< 0.01
	Present	44	16.1%	18	10.5%	15	18.8%	6	50.0%	5	45.5%	
Alcohol/ drugs abuse	Absent	256	92.8%	161	93.1%	73	91.3%	12	100%	10	90.9%	NS
	Present	20	7.2%	12	6.9%	7	8.8%	0	0%	1	9.1%	
Height	Height ≤ 2SD	10	3.6%	7	4.0%	2	2.5%	1	8.3%	0	0%	NS
	Normal	253	91.7%	155	89.6%	76	95.0%	11	91.7%	11	100%	
	Height ≥ 2SD	12	4.3%	10	5.8%	2	2.5%	0	0.0%	0	0%	
	Missing	1	0.4%	1	0.6%	0	0.0%	0	0.0%	0	0%	
Blood pressure	Low	12	4.3%	8	4.6%	2	2.5%	2	16.7%	0	0.0%	NS
	Normal	253	91.7%	159	91.9%	75	93.8%	9	75.0%	10	90.9%	
	High^c^	7	2.5%	4	2.3%	1	1.3%	1	8.3%	1	9.1%	
	Missing	4	1.4%	2	1.2%	2	2.5%	0	0.0%	0	0.0%	

NDD = Neuro Developmental Disorder. BMI = Body Mass Index. MPA = Minor Physical Anomalies

NS = Not Significant. SD = Standard Deviation

^a^ Underweight: BMI ≤ 5th percentile

^b^ Overweight, obese: according to the International Obesity Task Force[27]

^c^ Blood pressure high: measured 3 times

^*^Chi squared test. A p-value < 0.05 (two-sided) was considered statistically significant

Table 3 presents the laboratory findings of a subsample (n = 97). Vitamin D3 deficiency < 50 nmol/ L was present in 71 children (73.2%) and highly prevalent across all diagnoses (NDD 68.1%, affective disorders 79.5%, eating disorder 100% and psychosis 62.5%). Dyslipidemia (cholesterol, triglycerides, LDL-cholesterol and non-HDL cholesterol) was found in three children (3.1%); high levels of prolactin (> 500 mE/L) in four children (all of them using risperidone). For hyperprolactinemia, there was no macroprolactin in these four children. None of the children on antipsychotics had increased glucose levels.

**Table 3:** Laboratory findings of a subsample of children (n = 97)

		Total		NDD		Affective Disorder		Eating Disorder		Psychosis		p-value*	
		(n = 97)		(n = 47)		(n = 39)		(n = 3)		(n = 8)			
		n	%	n	%	n	%	n	%	n	%		
Vitamin D3	> 50nmol/L	23	23.7%	15	31.9%	8	20.5%	0	0%	3	37.5%	NA	
	< 50 nmol/L	71	73.2%	32	68.1%	31	79.5%	3	100%	5	62.5%		
Prolactinaemia	Normal	93	95.9%	44	93.6%	38	97.4%	3	100%	8	100%	NA	
	> 500 mE/L	4	4.1%	3	6.4%	1	2.6%	0	0%	0	0%		
Lipid spectrum^a^	Normal	94	96.9%	46	97.9%	37	94.9%	3	100%	8	100%	NA	
	Dyslipidemia^a^	3	3.1%	1	2.1%	2	5.1%	0	0%	0	0%		
Glucose	Normal	96	99.0%	47	100%	38	97.4%	3	100%	8	100%	NA	
	< 5.6mmol/l	1	1.0%	0	0%	1	2.6%	0	0%	0	0%		

NDD = Neuro Developmental Disorder

N/A = not applicable due to small numbers in multiple columns

^a^ Lipid spectrum comprised levels of cholesterol, triglycerides (TG), low-density lipoprotein (LDL) high-density lipoprotein (HDL) according to reference values for children.

^b^ Dyslipidemia was defined as total cholesterol > 5.2 mmol/L (200 mg/dl), LDL > 3.4 mmol/L (130 mg/dl), HDL < 0.9 mmol/L (35 mg/dl), or triglycerides > 1.7 mmol/L (150 mg/dl), or a combination thereof.

*Chi squared test

## Discussion

This study found high somatic comorbidities in a large naturalistic sample of 276 children with broad psychiatric diagnoses, including NDD, affective disorders, eating disorders and psychosis. The primary somatic comorbidities were (1) problems associated with food intake, including overweight, obesity and vitamin D3 deficiency and (2) sleeping problems, mainly insomnia. Our findings indicate that somatic comorbidities are widespread in children and adolescents with psychiatric disorders. Assessing various somatic comorbidities in a sample consisting of several child psychiatric disorders, allowed to see its broad variation including problems with weight, problems related to food intake, vitamin D3 deficiency (< 50nmol/l), insomnia and minor physical anomalies.

With respect to problems in weight 12.0% of our sample was obese compared to 2.5% in the general Dutch population in the same period of this study [31]. Similarly, the percentage of overweight was 19.9%, compared to 14.2% of children in the general Dutch population [32]. These percentages increased for children using antipsychotic medication (25% for overweight children and 13.5% for obese children). These findings are consistent with previous studies. For example, in children with NDD, prevalence rates of overweight vary between 11 and 34% (our study 20.2%), and for obese children, between 12 and 23% (our study 12.2%) [4, 5, 33–35].

A recent meta-analysis indicated that obese children are more at risk for depression than normal-weight children with a prevalence of 10.4% [36]. In the opposite direction, our results displayed a prevalence of 12.5% of obesity among children with depression and anxiety disorders (affective disorders). Likewise, the same symptoms related to lifestyles, such as sedentary habits, disordered sleep, insufficient physical activity and dysregulated food consumption, are common in obese children and children with affective disorders [37]. For an optimal development of children, it is essential to be aware of these symptoms and to address both psychiatric and somatic conditions. As obesity is an important risk factor for cardiovascular disease during later life, it is important to be aware of the risk factors in children, such as some types of medication, the psychiatric disorder itself, genetic variations with obesity and lifestyle related factors [38].

Furthermore, our study showed a prevalence of vitamin D3 deficiency (< 50nmol/l) in the whole group of children with psychiatric disorders of 73%. This is much higher compared to reported 30% in the general population of Dutch children [39]. Vitamin D3 deficiency is associated with a range of adverse somatic and psychiatric outcomes (cardiovascular, diabetes, cancer, depression, NDD, psychosis and dementia) and might be a risk factor for impaired brain development [40, 41]. Reversely, vitamin D3 deficiency could be due to unhealthy lifestyle such as poor diet quality, being overweight and reduced exposure to sunlight by an imbalance between indoor and outdoor activities, which may be more common in children with psychiatric disorders [39, 40]. Additionally, a recent study revealed an inverse association between BMI and vitamin D3 levels in children with ASD and internalizing disorders [42], which is in line with studies in the general population [43, 44]. However, the underlying associations of vitamin D3 deficiency in somatic and psychiatric disorders are still poorly understood and questions for supplementation and lifestyle intervention remain.

The next main finding is the high rate of insomnia in our population (66%) compared to primary school-aged children (5–30%) and adolescents (4–13%) in the general population [45]. Insomnia is the most common sleep disorder in children and is primarily characterized by difficulty initiating or maintaining sleep and/or poor sleep quality, which results in significant impairments in daytime functioning [46, 47]. Sleep problems are complex and viewed as part of neurologic and psychiatric disorders (e.g., ASD, affective disorders, epilepsy, psychosis, migraine) [45–47]. For some psychiatric disorders sleep problems are also part of the criteria itself (e.g., depression and anxiety disorders) [48].

Several studies show that estimates of insomnia are higher in pediatric populations with psychiatric diagnoses, including ADHD, ASD, anxiety and depression which is in line with our findings [49, 50]. For instance, our study found that insomnia in children with NDD was comparable with the existing literature (50–80%) [49, 51, 52]. Also, for insomnia in children with affective disorders, similar rates are found: in our study 81% compared to 75–90% for depression and anxiety in previous studies [48, 50, 53]. Notably, in case of insomnia, the symptoms of depression or anxiety disorder are even more severe [48, 50, 53]. In addition, cognitive behavioral therapy for insomnia provides a non-pharmacological option for improving sleep in psychiatric patients [18]. This is also relevant for children with psychiatric disorders in which the relationship between sleep and behavioral interventions has not been fully elucidated.

The clinical assessment further showed a high rate of three or more minor physical anomalies (MPA) in children with NDD (53%) and psychosis (55%), which is also consistent with the literature [54, 55]. While a single MPA is not uncommon in the general population, a greater number of MPAs is associated with somatic comorbidities and underlying genetic variations [56]. As such, careful analysis of the combination of NDD, childhood psychosis, somatic comorbidities and MPA requires an simultaneous assessment to consider referral to a clinical geneticist [14].

Besides the physical outcomes, 66% of the children in our study did have a primary caregiver with a psychiatric diagnosis and 56% had been exposed to stressful life events (SLE; divorce of caregivers, death of caregiver, serious illness to family members, physical and/or sexual abuse, bullying at school). The Netherlands Mental Health Survey and Incidence Study [57] revealed an estimation of 3.5% of Dutch children with a caregiver with a psychiatric diagnosis; the National Survey of Children’s Health in the United States estimated that 7.2% of the US children had at least one caregiver with poor mental health [58]. The higher rate of primary caregiver’s psychiatric diagnosis in this study may be explained by the included study population of children with psychiatric disorders recruited in a specialized, academic psychiatric hospital for children and adolescents. This may provide a selection of the population with higher rates of disorders in the family.

Stressful life events (SLE) are defined as negative life events or chronic adversities in the children’s school-, family- or inter-personal environment that fall outside an individual’s normative life experiences [59]. The reported prevalence of SLEs in children is high and varies between 40 and 80% in studies [59–61]. There is no single definition of SLE and the impact of an SLE may vary per event and child [60; 62]. In this study SLE meant divorce of caregivers, death of caregiver, serious illness to family members, physical and/or sexual abuse, bullying at school and living in foster care. Financial problems, family or employment conflicts of caregivers are also examples of SLE’s, but we did not take them into account due to the lack of data of these factors. Poor mental health among primary caregivers and exposure to SLE are associated with poor mental and physical health in children across the lifespan [57–62].

Unfortunately, accurate and timely somatic assessment in children with psychiatric disorders can be challenging due to atypical presentation of symptoms, difficulties in describing and/or expressing subjective experiences or symptoms and insufficient expertise about somatic comorbidities in this population [11]. In line with our study, Agnafors et al. (2019) showed associations between a wide range of psychiatric and somatic disorders across all types of conditions and across all ages in a register-based study [12]. This warrants the question to implement simultaneous assessment of somatic and psychiatric symptoms in children and adolescents with psychiatric diagnoses.

Among the strengths of our study is the extensive assessment of the somatic comorbidities of a relatively large number of children and adolescents with different psychiatric disorders. This showed a broad variation of comorbidities, underscoring the importance of routine somatic assessment in children with psychiatric disorders. Second, the somatic and psychiatric assessment was performed with great scrutiny and based on the assessment of two clinical experts (independent child and adolescent psychiatrists) and the psychiatric diagnoses were based on an evaluation by a multidisciplinary team. Additionally, the somatic assessments were carried out in a standardized way, allowing consistent and reliable collection of data. Third, we ran additional analyses with different variables to determine the influence of these variables (gender, age, medication and SES) on the physical examination and laboratory findings.

There are also several limitations regarding this study. First, lack of a matched control group may limit the specificity of our findings. However, prevalence rates from children from the general population were available. Given the remarkable differences with this norm population, our general concerns about the high rates of somatic concerns in children with psychiatric disorders appear valid. Second, all children in this study were recruited in a child psychiatric center, which may introduce ascertainment/selection bias. A third limitation is that, although we have included children with various child psychiatric disorders, the number of patients in some specific diagnostic categories was small(e.g. psychosis and eating disorders), not allowing additional analyses to explore potential relationships between specific diagnosis and somatic comorbidities. A fourth limitation is the cross-sectional design which does not allow for further exploration of intricate relationships between psychiatric and somatic concerns over time.

We recommend that future studies investigating physical health in child and adolescent psychiatric populations collect longitudinal data in large samples of children and typically developing peers, including those with intellectual disabilities. At a broader public health level, research is required into the effectiveness of interventions that promote a healthy lifestyle, especially in child psychiatric population. Also, the role of vitamin D3 in psychiatric and somatic diseases needs further exploration, for instance, how it relates to other health factors (such as BMI, age, gender, diet, ethnicity, season, etc.), but also what the effects of supplementation are in relation to diagnosis. Furthermore, research of underlying genetic variation may provide deeper insight into patient (sub)groups more susceptible to mental and physical health problems than others. Ultimately, this may guide personalized treatment approaches.

## Summary

In conclusion, the findings of this study underscore the high prevalence of somatic comorbidity in children and adolescents with psychiatric disorders and highlight the importance of standardized somatic screening in this group. The primary somatic comorbidities, including higher-than-healthy weight, vitamin D3 deficiency and sleep problems, may be strongly related to lifestyle factors and future studies may need to address this. As such, the findings of this study suggest that mental health professionals may need to asses and treat somatic comorbidities or refer for adequate treatment. Clinicians must address mental *and* physical health, to ensure maximal well-being and the best possible outcomes for children and adolescents with psychiatric illnesses.

## Data Availability

The datasets generated and/or analyzed during the current study are not publicly available due to ethical restrictions and personal data protection. However, reasonable requests for patient level data should be made to the corresponding author and will be considered after discussion with the ethical board. As far as possible, we intend to include all relevant data in the manuscripts.
